# Determinants of Generalized Anxiety Disorder Symptoms in Residents of Fort McMurray 12 Months Following the Devastating Flooding In 2020

**DOI:** 10.1192/j.eurpsy.2023.454

**Published:** 2023-07-19

**Authors:** E. Owusu, R. Shalaby, E. Eboreime, N. Nkire, M. A. Lawal, B. Agyapong, H. Pazderka, G. Obuobi-Donkor, M. K. Adu, W. Mao, F. Oluwasani, V. I. Agyapong

**Affiliations:** ^1^Department of Psychiatry, University of Alberta, Edmonton; ^2^Department of Psychiatry, Dalhousie University, Halifax, NS, Canada

## Abstract

**Introduction:**

The flood in Fort McMurray (FMM) which occurred between April 26 and May 2, 2020, is known to have displaced an estimated population of 1,500 people, and destroyed or damaged about 1,230 buildings. In all, it is estimated to have caused about $228 million in losses.

**Objectives:**

This study aims to identify the determinants of likely Generalized Anxiety disorder (GAD) among respondents 12 months after the 2020 flooding

**Methods:**

Data for the study were collected through a cross-sectional survey sent through REDCap and hosted online from the 24th of April to the 2nd of June 2021. The self-administered questionnaire was emailed to respondents using community, government, school, and occupational platforms. Demographic, flooding-related variables and clinical data were collected. A validated instrument, the GAD-7 was used to collect information on likely GAD. Consent was implied by completing the survey forms, and the University of Alberta Health Research Ethics Committee approved the study.

**Results:**

Of the 249 residents surveyed, 74.7% (186) respondents completed the online survey, 81.6% (80) were above 40 years, 71% (132) were in a relationship, 85.5% (159) were females, and 94.1% (175) were employed. The prevalence of likely GAD was 42.5% in our study. Predictors of likely GAD among respondents included positive employment status (OR = 30.70; 95% C.I. 2.183–423.093), prior diagnosis of depression (OR = 3.30; 95% C.I. 1.157–9.43), and the perceived need to have mental health counseling (OR = 6.28; 95% C.I. 2.553–15.45).

**Conclusions:**

This study showed that there was an increased magnitude of moderate to high anxiety symptoms among respondents following the natural disaster, particularly the flood in 2020. The predictors of likely GAD include positive employment status, a history of depression diagnosis, and the need to have mental health counseling. Policy formulators may reduce the risk of anxiety after flooding in vulnerable areas by addressing these factors.

**Disclosure of Interest:**

None Declared

